# Comparison of complications between reverse-tapered and nontapered peripherally inserted central catheters

**DOI:** 10.1371/journal.pone.0285445

**Published:** 2023-05-04

**Authors:** Hyun Soo Bae, Kun Yung Kim, Young-Min Han

**Affiliations:** 1 Department of Radiology, College of Medicine, Jeonbuk National University, Jeonju, South Korea; 2 Research Institute of Clinical Medicine of Jeonbuk National University–Biomedical Research Institute of Jeonbuk National University Hospital, Jeonju-si, Korea; Manchester University NHS Foundation Trust, UNITED KINGDOM

## Abstract

Purpose of this study was to compare the complication rates between reverse-tapered and nontapered peripherally inserted central catheters (PICCs). In total, 407 patients who had an inpatient clinic-based PICC insertion between September 2019 and November 2019 were retrospectively analyzed. Seven PICC types were used (4 reverse tapered: 4-Fr single-lumen (n = 75), 5-Fr single-lumen (n = 78), 5-Fr double-lumen (n = 62), and 6-Fr triple-lumen (n = 61); 3 nontapered: 4-Fr single-lumen (n = 73), 5-Fr double-lumen (n = 30), and 6-Fr triple-lumen (n = 23)). Complications such as periprocedural bleeding, delayed bleeding, inadvertent removal, catheter obstruction by thrombosis, infection, and leakage were investigated. The overall complication rate was 27.1%. The complication rate was significantly higher for nontapered PICCs than reverse-tapered PICCs (50.0% vs 16.7%, P < 0.001). The overall periprocedural bleeding rate was significantly higher for nontapered PICCs than for reverse-tapered PICCs (27.0% vs 6.2%, P <0.001). The overall inadvertent removal rate was significantly higher for nontapered PICCs than for reverse-tapered PICCs (15.1% vs 3.3%, P < 0.001). There were no other significant differences in complication rates. Nontapered PICCs were associated with higher rates of periprocedural bleeding and inadvertent removal than reverse-tapered PICCs.

## Introduction

Peripherally inserted central catheters (PICCs) are commonly used for patients in need of long-term intravenous therapy [1, 2]. However, PICC-related complications can adversely affect treatment outcomes. Several studies have reported that the complications could be related to the characteristics of PICCs. For example, increased PICC lumen number has been reported as a strong risk factor for CLABSI (central line associated bloodstream infection) [3–5]. The large diameter of PICCs has been reported to increase the incidence of deep vein thrombosis (DVT), which is another well-known PICC-associated complication [6, 7]. However, a recent randomized controlled trial reported that there was no association between PICC diameter and DVT [8].

PICCs are divided into two types: reverse-tapered and nontapered. With the reverse-tapered type, the catheter diameter gradually increases toward the hub of the PICC. A large diameter of the catheter at the puncture site might potentially reduce bleeding, but it could also result in the stagnation of blood flow, which could induce DVT. With the nontapered type, the catheter has an equal diameter from the tip toward the hub. The diameter of a nontapered PICC is usually smaller than the peel-away sheath; thus, puncture site bleeding might frequently occur. However, blood flow stagnation could be reduced. There is only one study comparing the two types of PICC. Itkin et al. reported that the catheter-related DVT rate was not significantly different between the two types of PICC [8]. However, only double-lumen catheters were used in their study. Furthermore, complications other than DVT were not evaluated. The aim of this study was to compare complication rates between reverse-tapered and nontapered PICCs.

## Materials and methods

### Patients

**Fig 1:**
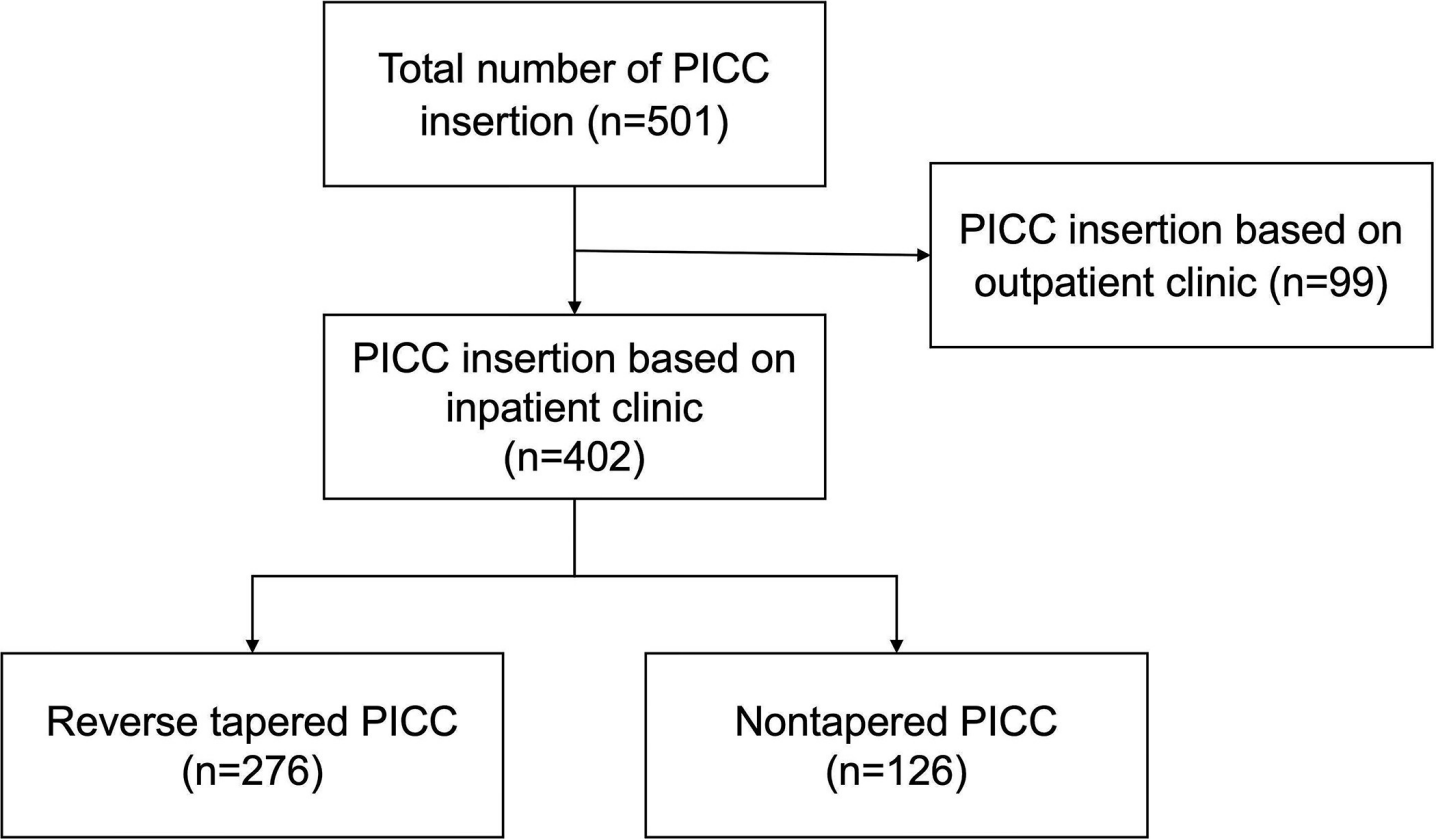
Inclusion and exclusion criteria.

**Fig 2:**
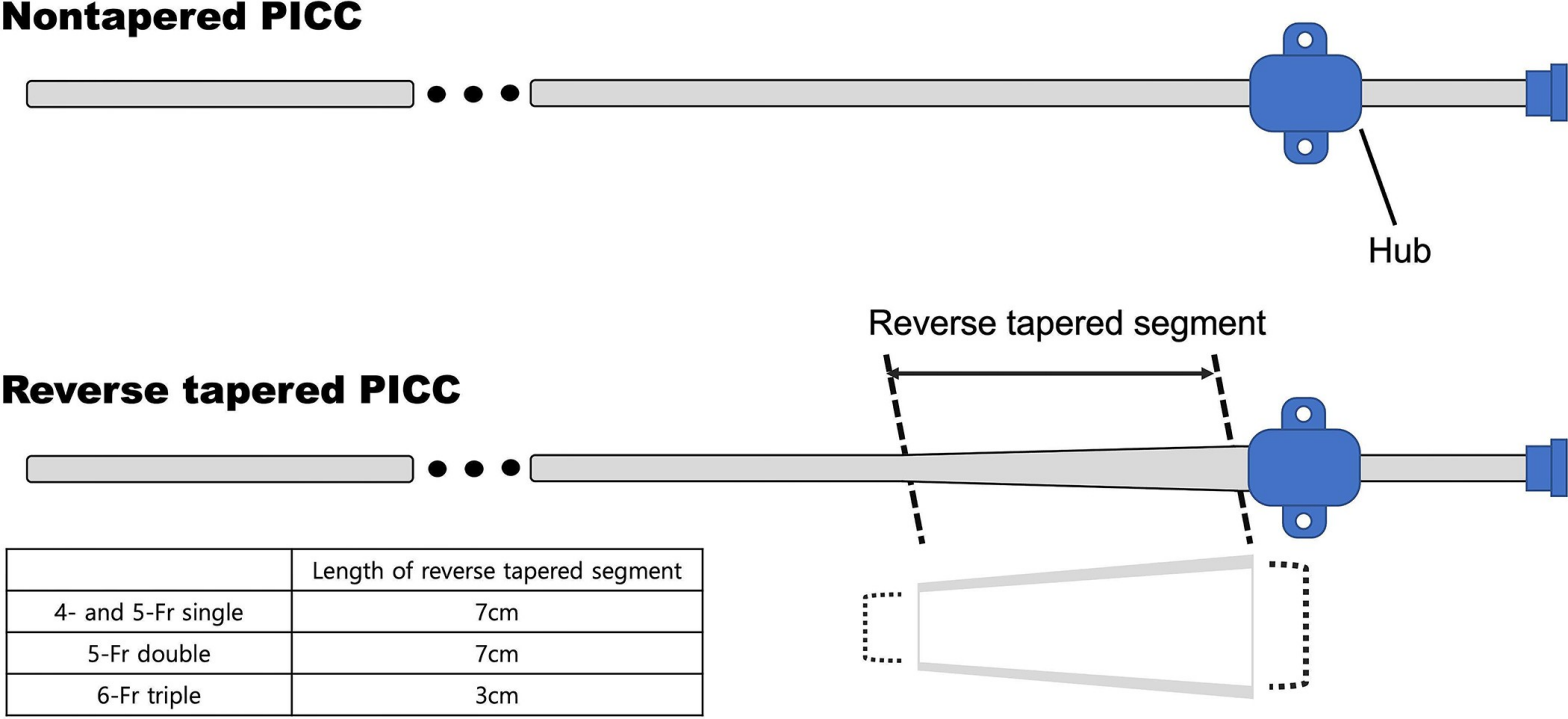
Illustration of nontapered and reverse-tapered PICCs. The diameters of nontapered PICC remain equal throughout the catheter. For a reversed-tapered PICC, the diameter of the catheter gradually increases over a distance of 7 cm or 2 to 3 cm from the hub.

This single-center, retrospective study was approved by the institutional review board. Informed consent was waived by the board because of the retrospective nature of the analysis. Data from September 2019 to November 2019 were reviewed. During this period, a total of 501 PICCs were used in 484 patients. Ninety-nine patients who underwent the procedure at an outpatient clinic were excluded due to limitations in follow-up (Fig 1). Thus, a total of 402 PICC insertions in 387 patients were analyzed in this study. Patients’ data are summarized in Table 1. The seven PICC types used in this study were as follows: 4- and 5-Fr single-lumen, 5-Fr double-lumen, and 6-Fr triple-lumen reverse-tapered PICC (Turbo-Ject® Power-Injectable PICC, Cook medical) and 4-Fr single-lumen, 5-Fr double-lumen, and 6-Fr triple-lumen nontapered PICC (Arrow® PICC, Teleflex). The length of reverse tapering was 7 cm in 4- and 5-Fr catheters and was 2 cm in 6-Fr catheters (Fig 2).

**Table 1:** Demographics of patients.

	Reverse-Tapered PICC (n = 276)	Nontapered PICC (n = 126)	P-value
Mean age	68.7 ± 14.0	71.0 ± 14.0	0.128
Sex			0.88
Male	143 (51.8)	63 (50.0)	
Female	133 (48.2)	63 (50.0)	
History of hypertension	152 (55.1)	70 (55.6)	0.986
History of diabetes	75 (27.2)	45 (35.7)	0.106
History of CVD	52 (18.8)	29 (23.0)	0.404
History of stroke	65 (23.6)	18 (14.3)	0.046
History of anticoagulation	60 (21.7)	24 (19.0)	0.629
Platelet (x10^3^)	238.19 ± 137.8	240.53 ± 129.3	0.872
PT (INR)	1.32 ± 0.44	1.26 ± 0.28	0.140
aPTT (seconds)	32.91 ± 7.82	33.80 ± 12.88	0.392
Side			0.858
Right	252 (91.3)	115 (91.3)	
Left	24 (8.7)	11 (8.7)	
Puncture vein			0.353
Basilic	240 (87.0)	109 (86.5)	
Brachial	32 (11.6)	17 (13.5)	
Cephalic	4 (1.4)	0 (0.0)	
PICC lumen			0.678
Single	4-Fr: 75 (27.2) 5-Fr: 78 (28.3)	73 (57.9)	
Double	62 (22.5)	30 (23.8)	
Triple	61 (22.1)	23 (18.3)	

* Numbers in parentheses are percentage

### PICC insertion procedure and follow-up

After skin sterilization and injection of local anesthetics, the basilic or brachial vein was punctured under ultrasound guidance. A guidewire was used for positioning the catheter tip under fluoroscopy. The preferable location of the catheter tip was the superior vena cava (SVC)–right atrial (RA) junction. The length between the puncture site and the SVC–RA junction was measured, and then the catheter was trimmed accordingly.

With nontapered PICC catheter insertion, the catheter was inserted without trimming, generally. The tip of the catheter was positioned in the SVC–RA junction first, then an external fixator was fitted into the catheter at the puncture site and fixed in the skin by applying suture or StatLock.

After the procedure the PICC insertion site was dressed by applying transparent film. Saline flushing after each use was done during the follow-up period. The follow-up endpoint was the time when the catheter was removed. Complications such as periprocedural bleeding, delayed bleeding, inadvertent removal, catheter obstruction by thrombosis, infection, leakage, and catheter patency were investigated.

### Definitions

Periprocedural bleeding was defined as bleeding that requires additional bleeding control such as manual compression at least 2 minutes right after the procedure. Delayed bleeding was defined as the occurrence of bleeding at least 1 hour after the procedure. Catheter obstruction was defined as an uncorrectable obliteration of the PICC lumen. In-body length was defined as the length of the catheter between the PICC tip, which is positioned at RA-SVC junction, and the hub of the PICC. Out-body length was defined as the length of the catheter from the hub of the catheter to the puncture site of the skin. Infection was defined as the occurrence of CLABSI according to the definition of The National Healthcare Safety Network surveillance [9, 10].

### Statistical analysis

Numerical variables were expressed as mean ± standard deviation. The Student’s t-test was used to compare continuous variables. The Chi-square test was used to compare categorical variables. To identify independent predictors of periprocedural bleeding and inadvertent removal, univariate and multivariate logistic regression analyses were performed. The variables considered were age, sex, history of diabetes, history of cerebrovascular disease, history of anticoagulants, platelets, prothrombin time (international normalized ratio, INR), type of PICC, number of lumen, diameter at hub, proportion of catheter to target vein diameter, side, punctured vein, diameter of punctured vein, and depth of punctured vein. The diameter and depth of the punctured vein were dichotomized by the cutoff value with the highest Youden index (14). Variables with a P value < 0.1 on univariate analysis were used for multivariate analysis. A two-sided *P* value < 0.5 was considered statistically significant in the final model. All statistical analyses were performed using MedCalc version 20.109 (Foundation for Statistical Computing, Vienna, Austria).

## Results

The overall complication rate was 27.1% (109 of 402 patients). The complication rate was significantly higher for nontapered PICCs (50.0%, 63 of 126 patients) than for reverse-tapered PICCs (16.7%, 46 of 276 patients) (P < 0.001). The periprocedural bleeding rate was significantly higher for nontapered PICCs (27.0%, 34 of 126 patients) than for reverse-tapered PICCs (6.2%, 17 of 276 patients) (P <0.001). The inadvertent removal rate was significantly higher for nontapered PICCs (15.1%, 19 of 126 patients) than for reverse-tapered PICCs (3.3%, 9 of 276 patients) (P < 0.001). No other complication rates showed significant differences between the PICC types. Data are summarized in Table 2.

**Table 2:** Complication rates.

	Overall			Single lumen (4 Fr)			Double lumen (5 Fr)			Triple lumen (6 Fr)		
	Tapered (n = 276)	Nontapered (n = 126)	P-value	Tapered (n = 153)	Nontapered (n = 73)	P-value	Tapered (n = 62)	Nontapered (n = 30)	P-value	Tapered (n = 61)	Nontapered (n = 23)	P-value
Periprocedural bleeding	17 (6.2)	34 (27.0)	<0.001	6 (3.9)	14 (19.2)	<0.001	2 (3.2)	11 (36.7)	<0.001	9 (14.8)	9 (39.1)	0.033
Inadvertent removal	9 (3.3)	19 (15.1)	<0.001	2 (1.3)	11 (15.1)	<0.001	7 (11.3)	2 (6.7)	0.745	0 (0.0)	6 (26.1)	<0.001
Catheter obstruction	7 (2.5)	5 (4.0)	0.641	4 (2.6)	4 (5.5)	0.481	3 (4.8)	1 (3.3)	0.831	0 (0.0)	0 (0.0)	-
Infection	11 (4.0)	5 (4.0)	0.790	4 (2.6)	3 (4.1)	0.845	3 (4.8)	0 (0.0)	0.549	4 (6.6)	2 (8.7)	0.892
Leakage	1 (0.4)	0 (0.0)	0.687	0 (0.0)	0 (0.0)	-	0 (0.0)	0 (0.0)	-	1 (1.6)	0 (0.0)	0.670
Delayed bleeding	1 (0.4)	0 (0.0)	0.687	0 (0.0)	0 (0.0)	-	1 (1.6)	0 (0.0)	0.709	0 (0.0)	0 (0.0)	-

* Data from 5-Fr single lumen PICC (Cook) are omitted in this table. Total of 78 PICCs; periprocedural bleeding, 0 PICCs (0.0%); inadvertent removal, 1 PICCs (1.3%); catheter obstruction, 2 PICCs (2.6%); infection, 4 PICCs (5.1%); leakage, 0 PICCs (0.0%); delayed bleeding, 0 PICCs (0.0%)

* Numbers in parentheses are percentages

Nontapered type, triple lumen, diameter at hub, and more than 50% of catheter diameter to target vein diameter proportion were significant predictors of periprocedural bleeding (Table 3). Among these factors, multivariate analysis indicated that nontapered type (OR, 6.257; 95% CI 3.271–11.968; P < 0.001) and triple lumen (OR, 2.929; 95% CI, 1.474–5.822; P = 0.002) were independent risk factors for periprocedural bleeding.

**Table 3:** Univariate analysis of predictors of periprocedural bleeding.

Variables	Frequency	Periprocedural bleeding		P-value
		Yes	No	
Total number of PICC on inpatient clinic	402	51 (12.7)	351	-
Age ≥ 65	285	40 (14.0)	245	0.208
Male sex	206	23 (11.2)	183	0.348
History of hypertension	222	31 (14.0)	191	0.393
History of diabetes	120	19 (15.8)	101	0.218
History of stroke	81	9 (11.1)	72	0.634
History of anticoagulants taking	84	9 (10.7)	75	0.542
Platelet<100,000	62	8 (12.9)	54	0.956
INR>1.4	75	10 (13.3)	65	0.852
Nontapered type	126	34 (27.0)	92	<0.001
Number of lumen				
Single	226	20 (8.8)	206	-
Double	92	13 (14.1)	79	0.165
Triple	84	18 (21.4)	66	0.004
Diameter at hub				0.001
4-Fr (A1)	73	14 (19.2)	59	-
5-Fr (A2)	30	10 (33.3)	20	-
6-Fr (A3 and C1 4-Fr)	98	15 (15.3)	83	-
7-Fr (C2 and C1 5-Fr)	140	4 (2.9)	136	-
8-Fr (C3)	61	8 (13.1)	53	-
Catheter to target vein diameter proportion > 50%	155	12 (7.7)	143	0.021
Left side PICC	35	2 (5.7)	33	0.210
Punctured vein				
Basilic vein	349	40 (11.5)	309	-
Brachial vein	49	11 (22.4)	38	0.035
Cephalic vein	4	0 (0)	4	0.998
Vein diameter ≥ 4.48mm	204	25 (12.3)	179	0.792
Vein depth ≥ 5.93mm	201	22 (10.9)	179	0.296

* Numbers in parentheses mean percentages

*A1, Arrow single lumen; A2, Arrow double lumen; A3, Arrow triple lumen; C1, Cook single lumen; C2, Cook double lumen; C3, Cook triple lumen

History of anticolagulants, nontapered type, diameter at hub, and more than 50% of catheter diameter to target vein diameter proportion were predictors of inadvertent removal (Table 4). Among these factors, multivariate analysis indicated that nontapered type (OR, 5.237; 95% CI 2.283–12.011; P < 0.001) was independent risk factors for inadvertent removal.

**Table 4:** Univariate analysis of predictors of inadvertent removal.

Variables	Frequency	inadvertent removal		P-value
		Yes	No	
Total number of PICC on inpatient clinic	402	28 (7.0)	374	-
Age ≥ 65	285	21 (7.4)	264	0.621
Male sex	206	14 (6.8)	192	0.891
History of hypertension	222	15 (6.8)	207	0.855
History of diabetes	120	9 (7.5)	111	0.784
History of stroke	81	5 (6.0)	78	0.426
History of anticoagulants taking	84	1 (1.2)	83	0.047
Platelet<100,000	62	3 (4.8)	59	0.478
INR>1.4	75	2 (2.7)	73	0.123
Nontapered type	126	19 (15.1)	107	<0.001
Number of lumen				
Single	226	13 (5.8)	213	-
Double	92	9 (9.8)	83	0.204
Triple	84	6 (7.1)	78	0.651
Diameter at hub				0.009
4-Fr (A1)	73	9 (12.3)	64	
5-Fr (A2)	30	3 (10.0)	27	
6-Fr (A3; C1 4-Fr tip)	98	8 (8.2)	90	
7-Fr (C2; C1 5-Fr tip)	140	7 (5.0)	133	
8-Fr (C3)	61	1 (1.6)	60	
Catheter to target vein diameter proportion > 50%	155	5 (3.2)	150	0.026
Left side PICC	35	0 (0.0)	35	0.998
Punctured vein				
Basilic vein	349	26 (7.4)	323	-
Brachial vein	49	2 (4.1)	47	0.396
Cephalic vein	4	0 (0.0)	4	0.998
Vein diameter ≥ 4.48mm	204	14 (6.9)	190	0.935
Vein depth ≥ 5.93mm	201	11 (5.5)	190	0.243

* Numbers in parentheses mean percentages

*A1, Arrow single lumen; A2, Arrow double lumen; A3, Arrow triple lumen; C1, Cook single lumen; C2, Cook double lumen; C3, Cook triple lumen

Multivariate analysis indicated that nontapered type (OR, 6.257; 95% CI 3.271–11.968; P<0.001) and triple lumen (OR, 2.929; 95% CI, 1.474–5.822; P = 0.002) were independent risk factors for periprocedural bleeding.

## Discussion

Previous reports on the characteristics of PICCs have focused on clinical outcomes. Most of those reports primarily analyzed the risk of DVT. Grove et al. suggested that a catheter should be as small as possible to reduce the incidence of DVT, and Evans et al. reported that a large catheter size was related to an increased risk of DVT [6, 7]. Itkins et al. compared the DVT rate between reversed-tapered and nontapered PICCs in their randomized controlled trial. They reported that there was no significant difference between the two types of PICC [8]. In this study, we showed that PICC type is associated with certain complications. The rates of periprocedural bleeding and inadvertent removal were significantly higher for nontapered PICCs, while other complications were not significantly different between reversed-tapered and nontapered PICCs.

Periprocedural and delayed bleeding rates for PICCs have not been reported in previous studies. In our study, the periprocedural bleeding rate was significantly higher for nontapered PICCs than for reverse-tapered PICCs (27% vs. 6.2%, p < 0.001). The factors associated with an increased risk of periprocedural bleeding were nontapered type and triple lumen catheter. Considerable variety in periprocedural bleeding rate was shown among the catheters with a different diameter at the hub (2.9%– 33.3%). A catheter to target vein diameter > 50% was also associated with the decreased risk of periprocedural bleeding in univariate analysis. These results implied that the catheter profile is closely associated with periprocedural bleeding. With nontapered PICCs, the diameter of the peel-away sheath is usually larger than the diameter of the hub. Thus, bleeding could occur through the gap between the PICC catheter and dilated puncture site. Most periprocedural bleeding can be managed simply by manual compression, but an encircling suture around the catheter may be needed if there is persistent bleeding.

Another complication associated with catheter type was inadvertent removal. Our results showed a significantly higher inadvertent removal rate in nontapered PICCs compared to reverse-tapered PICCs (15.1% vs 3.3%, p < 0.001). This may be attributable to the difference in the method of PICC fixation. In nontapered PICCs, the catheter is introduced until its tip is in the SVC-RA junction without the trimming of its tip; then, an external skin fixator is assembled to the catheter according to the length inside the body. This method contributed to the reduction of procedure time. However, the external length of the catheter could be longer; consequently, the chance of accidental inadvertent removal may increase. Only simple catheter fixation methods such as StatLock and/or suture were used in this study. Advanced catheter securement system such as SecurAcath (Interrad Medical, Plymouth, MN, USA) might be considered to prevent inadvertent removal when nontapered PICC is used without trimming.

DVT is a recognized complication of PICCs. There was a concern that the reversed-tapered design hampers blood flow at the insertion site, which can result in DVT. However, a previous RCT showed the tapered design did not increase the number of DVTs, as had been hypothesized [8]. In this study, only DVTs that caused catheter obstruction have been documented due to the retrospective nature of this study. The rate of catheter obstruction was not significantly different between PICC types in this study. Although asymptomatic DVT may be underestimated, our results demonstrated a clinically relevant thrombosis rate causing catheter obstruction.

There were limitations in this study. First, this study was a retrospective, single-center study over a relatively short period of investigation. Second, the procedure was performed by multiple radiologists with different levels of experience. Although all participating radiologists received education about the definition of periprocedural bleeding and used the same technique in PICC insertion, there is inevitable bias from a technical perspective. Third, the hub of the nontapered PICC catheter was incompletely inserted. This might result in a significantly higher inadvertent removal rate in nontapered PICCs; thus, delayed complications such as infection or catheter obstruction might be underestimated. Fourth, the DVT occurrence rate was not analyzed in this study. As there was a previous RCT on DVT incidence, this study focused on other possible complications of PICCs. A future prospective study to compare complications between reverse-tapered and nontapered PICCs is warranted.

## Conclusions

In conclusion, nontapered PICCs were associated with higher rates of periprocedural bleeding and inadvertent removal than reverse-tapered PICCs. The reverse-tapered design does not appear to increase complication rates such as catheter obstruction by thrombosis. Collectively, it appears that nontapered PICCs do not offer specific advantages over reverse-tapered PICCs relative to complications.
